# Childbearing intention and its associated factors: A systematic review

**DOI:** 10.1002/nop2.849

**Published:** 2021-03-11

**Authors:** Mozhgan Hashemzadeh, Mohammad Shariati, Ali Mohammad Nazari, Afsaneh Keramat

**Affiliations:** ^1^ Student Research Committee School of Nursing and Midwifery Shahroud University of Medical Sciences Shahroud Iran; ^2^ Department of Community Medicine Tehran University of Medical Sciences Tehran Iran; ^3^ School of Nursing and Midwifery Shahroud University of Medical Sciences Shahroud Iran; ^4^ Center for Health Related Social and Behavioral Sciences Research Shahroud University of Medical Sciences Shahroud Iran

**Keywords:** childbearing intentions, ecological model, effective factors, systematic review

## Abstract

**Aim:**

This study aimed to provide comprehensive information about the core determinants of fertility intentions.

**Design:**

Systematic review.

**Methods:**

Ovid, MEDLINE, EMBASE, PsycINFO, CINAHL, Web of Science, SCOPUS and GOOGLE SCHOLAR were searched for the relevant articles published from 1946–December 2017. We updated our records by searching three computerized databases (Ovid MEDLINE, SCOPUS and WOS) from 2018–January 2021.

**Results:**

53 studies included in the qualitative synthesis. The results of some studies indicated the impact of demographic factors, physical and psychological health, happiness and child desire. The most frequent variables in a couple's mesosystem were marital status, parity, partnership satisfaction and gender role attitude. The mesosystem of childbearing intention also included family and peers network. The EXEO system of the ECSM includes certain variables, such as job characteristics, urban residence, housing condition. The macrosystem comprises cultural and societal principles with broader influences on the couple's system.

## 1 INTRODUCTION

Today, contraception use has changed the prospect of parenthood to a personal choice (Mills, 2011). In the early 19th century, the modern fertility transition began in France and the United States. (Gutmann & Fliess, 1993; Westoff, ) and quickly expanded across Europe (Westoff, 2015). The regulation of fertility was achieved by widely using contraception and abortion. In every case, the small family size represented the intent of the individual couple (Watkins, 1987). The practice of family limitation started to spread among the population in Europe (Knodel, 1977; Watkins, 1987). In this respect, the modern fertility transition appeared to result from the spread of innovative behaviour and not an adjustment to new socio‐economic circumstances. (Watkins, 1987). This European pattern quickly became popular in developed Asian and American societies (Beaujolai& Sobotka, 2019; Caldwell & Caldwell, 2005; Knodel, 1977; Sobotka, 2017). At the same time, the childbearing rate among less developed countries showed a significant reduction (Bongaarts, 2008; Timæus & Moultrie, 2020).

Low fertility and population ageing in most European countries (Fahlén, 2013) have attracted researchers’ and policymakers’ attention. Factors that build fertility intentions can explain variations in fertility changes cross‐nationally and over time (Harknett et al., 2014).

A large body of research explains possible reasons for low fertility, such as the economic situation, child‐rearing costs, women's education and employment (Cooke, ; McDonald, 2000; Mills et al., 2008). Since the 1990s, some recent studies have tried to explain fertility levels through gender (Yu & Kuo, 2017).

The literature suggests that childbearing decision‐making is a complex process involving many social, economic, political and individual factors. These include the availability of qualified and affordable childcare support, cultural norms, individual beliefs and partner suitability (Abma & Martinez, 2006; Clarke & Hammarberg, 2005; Cooke et al., 2010; Mills et al., 2011; Proudfoot et al., 2009).

Bronfenbrenner provides an ecological model that envisages the existence of several environments or contexts that may be analysed from four levels, which are all part of the same reality.

- The “microsystem” includes the roles, relationships, and activity patterns developed by a person in their relationship with their environment.
- The “mesosystem” or the relation between two or more microsystems in which the person is actively involved.
- The “exosystem” or those environments in which the person in the process of becoming is not so actively involved in but do affect his/her development.
- The “macrosystem” as the relationships, both in form and content of the lower order systems (micro‐, meso‐ and exo‐) that exist or may exist at the sub‐culture level or the culture as a whole, together with any belief system or ideology that supports these correlations (Rothbaum et al., 2002).

Applying ecological approaches is particularly suitable for factors associated with childbearing intentions (Balbo et al., 2013). The decision to have a child is a complex process involving many social, economic, political and individual factors. So it is better to describe it as a new system (Bronfenbrenner, 1994).

There are several reviews conducted about fertility (Balbo et al., 2013; Butler, 2004; Caldwell & Schindlmayr, 2003; Mills et al., 2011; Morgan & Taylor, 2006; Sobotka, ) that provide important views. Some of these studies have focused on specific aspects or geographical areas. As the predictors of fertility intentions are similar to the predictors of actual births (Harknett et al., 2014; Kuhnt & Trappe, 2016). A couple is the most important context for investigating fertility decision‐making (Spéder & Kapitány, 2009).

Our study focuses on the determinants of childbearing plans to provide a lens for understanding fertility behaviour in couples.

## 2 METHODS

This systematic review was carried out following PRISMA guidelines. Note that PRISMA is an evidence‐based minimum set of items for reporting in systematic reviews and meta‐analyses (For more information, see: www.prisma‐statement.org).

### 2.1 Search strategy

**FIGURE 1:**
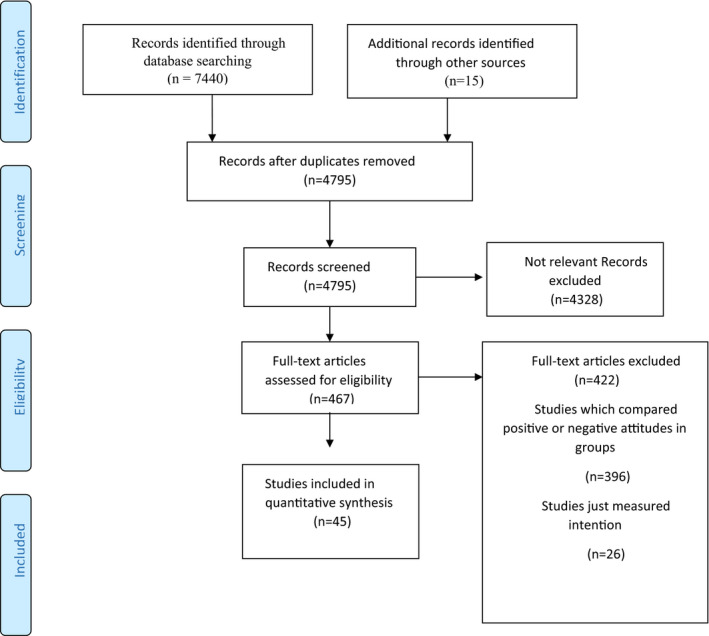
Flow chart of study selection

This systematic literature review was performed using electronic databases such as Ovid MEDLINE, EMBASE, PsycINFO, CINAHL, Web of Science, SCOPUS and GOOGLE SCHOLAR. The article search was performed with an alternate combination (and/or) of these search terms: “fertility”, “desire”, “intention”, “childbearing”, and “reproductive decision making”. The search was conducted from 1946 to December 2017 (see Appendix S1). Systematic reviews and meta‐analyses were manually checked to distinguish the related studies missed by electronic databases search. Figure 1

This study was the first step of a Ph.D. thesis, conducted in 2017. We updated our records by searching three computerized databases (Ovid MEDLINE, SCOPUS and WOS) from 2018 to January 2021.

### 2.2 Inclusion and exclusion criteria

We included the original studies in English, published in peer‐reviewed journals. As we wanted to study fertility in a family structure, we excluded adolescent fertility and included studies in which their participants were men and/or women of reproductive age (men aged 18–55 and women aged 18–45). Family planning methods were available in the reference country. The intention of fertility makes sense when people have access to family planning methods and have voluntary childbearing. Studies attributed to China were excluded due to child‐restricting national laws.

### 2.3 Screening

Two authors independently searched and screened studies for the inclusion criteria. A total number of 7,440 articles were identified and imported to Endnote X8. We removed 2,640 duplicated articles and screened titles and abstracts of the 4,795 ones. 467 relevant articles were fully assessed for more screening. We evaluated the eligibility of these articles and, finally, 45 studies were included. Any disagreements between the two authors were discussed until consensus was reached.

A total number of 1594 articles were identified through an updated search in 2021. After removing duplicated articles, titles, and abstracts of 1,041, articles screened. 72 relevant articles were fully assessed and 8 studies were included. Finally, 53 studies were included in this review. Table 1 provides information about the included studies.

**TABLE 1:** Information of included studies

Author	Year	Country	Target	Sample	Method	Dependent Varriables
Aassve, A. Arpino Bruno. Balbo Nicoletta	2016	Britain	Women & Men	1,463	Longitudinal	Subjective well‐being Happiness
Averett, S. L	2001	US	Women	4,679	Longitudinal	Age Maternity leave
Barber, et al	2019	US	Women	895	longitudinal	Couple Relationship
Bernhardt, E., et al	2016	Sweden	Men	2,273	longitudinal	Gender roll attitude Partnership status
Berninger, I. Weiß, B. Wagner, M	2011	Germany	Men &women	641	Cross‐sectional	Age Employment and Income partnership quality
Boivin, J., Buntin, L., & Kalebic, *N*	2018	79 countries	Men &women	10,045	Cross‐sectional	Desire for children Economic conditions physical health Social status of parents Child cost personal readiness
Bühler, C. and E. Fratczak	2007	Poland	Men &women	758	Cross‐sectional	Network partners
Brauner‐Otto, S. R. and C. Geist	2017	US	Men &Women	1,465	Longitudinal	Economic insecurity Education Employment and Income Race Gender
Cranney, S.	2015	Slovenia & Czech Republic	Women	5,453	Cross‐sectional	Religion Belief in God
De Wachter, D. & K. Neels,	2011	Belgium	Women	957	Cross‐sectional	Marital Status Parity Education Employment
Dommermuth. et al	2015	Norweg	Men &Women	1537	Cross‐sectional	
Fan, E & P. Maitra	2012	Australia	Men &women	19,914	Longitudinal	Men and women Desire effect Baby bonus
Fahlén, S	2013	10 European countries	Women	3,184	Cross‐sectional	Job security Family Support policies
Fiori F	2011	Italy	Women	5,143	Cross‐sectional	Income effect Job characteristic Division of domestic work
Fiori F, et al	2013	Italy	Women	15,870	Cross‐sectional	Age Marital status Employment Education Family network Area Economic insecurity
Goldscheider, F., et al.	2013	Sweden	Men &Women	1,096	Longitudinal	Marital status Gender roll attitude Division of domestic work Sharing child care tasks
Hanappi,D, et al	2017	Swiss	Women & Men	1634	longitudinal	Employment
Harknett, K. Billari, F. C. Medalia, C	2014	20 European countries	Women	7,436	Cross‐sectional	Labor market Division of domestic work Family support Policies
Kaufman, G.	2000	US	Women & Men		longitudinal	Gender roll attitude
Hayford, & Morgan	2008	US	Women	1,354	Cross‐sectional	Religion
Kjerulff H. Kristen	2013	us	Women	3,006	Longitudinal	Mode of First Delivery
Kim, E. H. W.	2017	Korea	Women	2,239	Longitudinal	Division of domestic work Informal and formal help in domestic labor
Kuhlmann, et al	2019	Honduras	women	6,629	Cross‐sectional	Intimate partner violence
Kuhnt, A. K. and H. Trappe	2016	Germany	Women & Men	4,881	longitudinal	Relationship Stability Employment Religion
Kulu Hill. Vikat Anderes	2007	Finland	Women	35,391	Longitudinal	Housing type
Meggiolaro, S.	2011	Italy	Women	790	Cross‐sectional	Age Parity Marital length context
Miettinen, A., et al	2011	Finland	Men &Women	2,143	Cross‐sectional	Gender roll attitude Education
Metcalfe, A., et al	2014	Canada	Women	835	Cross‐sectional	Work place support
Mills M, et al	2008	Dutch	Men &Women	3,458	Cross‐sectional	Education Employment Division of domestic work
Modena, F. & F. Sabatini	2012	Italy	Men &Women	19,551	Cross‐sectional	Age Parity Education Employment
Mynarska, M., & Rytel, J.	2020	Poland	Men & Women	939. Wome 470 men	Cross‐sectional	Joys of pregnancy, birth, & infancy Satisfaction of child‐rearing Negatives of childcare Discomforts of pregnancy and delivery Fears and worries of parenthood
Neyer, G., et al.	2013	10 European countries	Men &Women	44,630	Cross‐sectional	Employment Division of household & care (Gender Equity) Couple Satisfaction
Park, S. M., et al	2008	Korea	Women	2,211	Cross‐sectional	Age Parity Employment Social group Network Housing type
Park, S. M. & S. I. Cho	2011	Korea	Women	723	Cross‐sectional	Age Education value of child Family Support policies
Preis, H., et al	2020	Israil	Pregnant women	1,163	Longitudinal	Religion Education Negative birth experience
Rajan, S., et al	2018	India	Women	14,043	Cross‐sectional	Sex preference
Raymo, J. M., et al	2010	Japan & Italy	Women	8,299	Cross‐sectional	Intergenerational co‐residence
Riederer, B., & Buber‐Ennser, I.	2019	11 European countries	Men &women	10,137	Longitudinal	Regional context Rural and urban context
Rijken Æ et al	2009	Netherlands	Men &women	669	Longitudinal	Age Married status parity Partner relationship Quality
Risse, L	2010	Australia	Women	13,969	longitudinal	Educational level Parity Employment Income Baby bonus
Rosina, A. & M. R. Testa	2009	Italy	Women & Men	1,083	Cross‐sectional	Marital status Religion Couple agreement Division of domestic work
Schaffnit, S. B. & R. Sear	2017	Netherlands	Men &Women	2,288	Longitudinal	Family support Environment
Spéder, Z. & B. Kapitány	2009	Hungary	Men &Women	4,471	Longitudinal	Age Marital status Parity Education Employment Religion Couple Satisfaction
Sinyavskaya &. Billingsley	2013	Russia	Women	5,622	Cross‐sectional	Employment
Testa, M. R., et al	2011	Italy	Men &women	2,356	Longitudinal	Age Couple agreement
Testa, M. R., et al.	2012	Australia	Women	3,402	Cross‐sectional	Couple agreement
Testa, M. R	2014	27 European countries	Women	9,452	Longitudinal	Education
Vignoli, D., Mencarini, L., & Alderotti, G	2020	22 countries	Women & Men	10,565	Longitudinal	Subjective well‐being Job security Life satisfaction
Vignoli, D., et al	2012	Italy	Women	50,000	Cross‐sectional	Age Partnership status Religiosity Region Educational level Economy and Employment Housing condition
Vitali, A., et al.	2009	11 European countries	Women	5,529	Cross‐sectional	Family orientation Work orientation
Wesolowski, K.	2015	Ukraine	Women	749	Cross‐sectional	Age Education Partnership status Individual value Availability of child care Importance of Environmental Pollution and health concerns
Yoon, S. Y	2016	Korea	Women	235	Longitudinal	Gender equality Division of house work and child care
Yu, W. H. & J. C. L. Kuo	2017	Japan	Women & Men	1964	Cross‐sectional	Employment Economic condition Job security

### 2.4 Quality assessment

We used the STROBE checklist, which consisted of 22 items with a maximum score of 30. Studies with more than 23 total points were considered as high quality and those with less than 16 points were considered as low quality. Studies that received a total point between 16 and 23 were considered as medium quality. Among 53 studies, 46 (87%) were classified as high and 7 (13%) were classified as moderate. So, all the studies were included in our review. Among the 7 articles with medium quality, five got 23 points, one got 22 points, and the last one got 20 points. None of the articles had the main weakness in the methodological or result area. Appendix S2 provides a quality assessment of the included studies.

### 2.5 Data extraction

The following variables were extracted from the included studies: author's name, year of publication, country, study‐design, sample‐size, statistical analysis method, scale and primary results (variable associating childbearing).

#### 2.5.1 Applying Bronfenbrenner's ecological model to the childbearing intention

**FIGURE 2:**
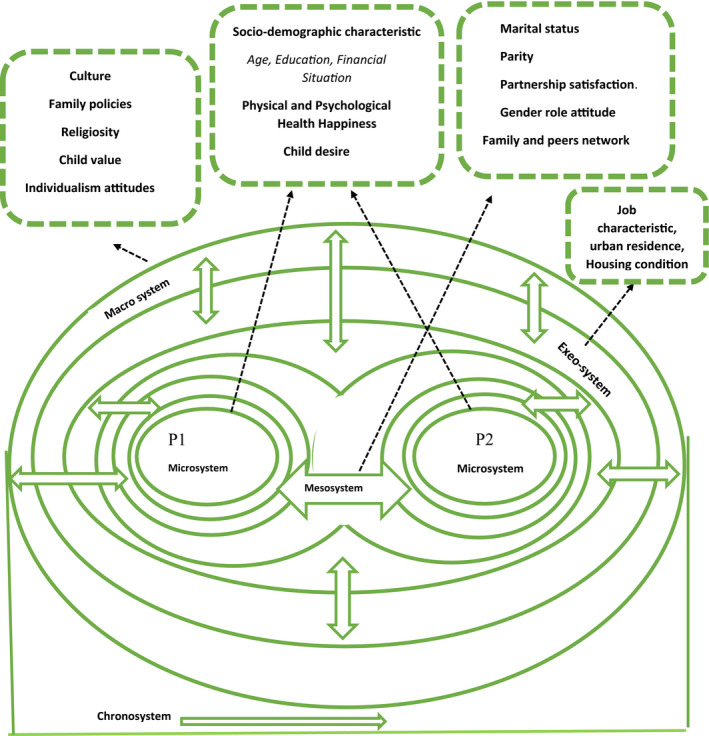
Ecological couple system model (ECSM)

A new concept was developed in which a couple's system acts like a union system in dyadic exclusivity. Based on this hypothesis and the overview of primary studies, a union system comprising two microsystems (individual and his or her partner) was developed, The ecological couple system model (ECSM) is a conceptual model of associated factors with fertility intention as a union system that interacts with all different systems in ecological systems theory. Other systems (mesosystem, exosystem, macrosystem and chronosystem) affect the ECSM (Figure 2).

## 3 RESULTS

53 Studies included in the qualitative synthesis. The characteristics of the included studies are shown in Table 1. According to the ecological model, 32 studies (60.4%) were based on Microsystem variables, 39 (73.6%) used mesosystem variables, 30 (56.6%) used EXEO system variables, 26 (49.1%) used Macrosystem variables, 5 (9.4%) used CHRONO system variables and 36 (67.9%) included variables from 2 or more levels.

We next describe the factors associated with childbearing intention through Bronfenbrenner's ecological systems theory.

### 3.1 Microsystems

The closest level to the individual is the microsystem. Numerous microsystems form throughout an individual's life. The microsystem of the ECSM includes information about socio‐demographic characteristics, physical and psychological health, happiness and child desire.

#### 3.1.1 Socio‐Demographic Characteristics

Socio‐demographic characteristics include age, education and financial situation.

##### Age

Age affects fertility intention both in men and women. Undoubtedly, the age of the woman has an essential role in fertility intention (Berninger et al., 2011; Fiori et al., 2013; Hanappi et al., 2017; Park et al., 2008; Risse, 2010; Wesolowski, 2015). The older the woman is at the beginning of the cohabitations, the sooner the couple has their first child. For the second and the third child, the age of the woman has a negative influence (Rijken & Liefbroer, 2009).

##### Education

In most developed countries, the educational level of couples has a positive influence on their fertility intentions (Testa, 2014) This result is similar in different age groups (De Wachter & Neels, 2011; Dommermuth, et al., 2017; Fiori et al., 2013; Goldscheider et al., 2013; Spéder & Kapitány, 2009; Wesolowski, 2015).

##### Financial situation

The birth requires access to financial resources. Better career prospects increase childbearing desire in both men and women (Bühler & Fratczak, 2007; Kuhnt & Trappe, 2016; Sinyavskaya & Billingsley, 2015; Yu & Kuo, 2017). Household income plays a key role in childbearing intentions (Hanappi et al., 2017). The intention of the second child is lower among families with limited income (Fiori, 2011).

#### 3.1.2 Physical and psychological health

Health concerns can seriously affect the second child's intentions among couples (Boivin et al., 2018). Having a negative birth experience could adversely affect women's fertility intentions. (Preis et al., 2020). The mode of the first delivery can affect childbearing intention in women. The mode of delivery can affect the next child's intention. The tendency to have two or three children is less in women who had a caesarean delivery (CD) compared with those who had a vaginal delivery (Kjerulff et al., 2013).

#### 3.1.3 Happiness

Happier men and women prefer to become parents sooner (Spéder & Kapitány, 2009). Happiness has different effects on childbearing intentions. Women's happiness seems to matter more for second child decision‐making. (Aassve et al., 2016). Optimistic people who are most satisfied with their life course and their prospects are more likely to realize their fertility intentions (Spéder & Kapitány, 2009).

#### 3.1.4 Child desire

Fertility preference is an important predicting factor for childbearing intentions. The possibility for an additional child is 83% higher when the wife's desire is higher and 48% lower when her desire is lower (Fan & Maitra, 2010). So, in predicting the birth outcomes, the wife's tendency has a higher effect (Fan & Maitra, 2010; Testa, 2012).

Among women, two positive motives, “feeling needed and connected” and “joys of pregnancy, birth, and infancy” were identified as being predictive of stronger childbearing desire. Among men, the significant predictors of stronger childbearing desire were “satisfaction of child‐rearing” and “traditional parenthood” (Mynarska & Rytel, 2020).

In societies like India, the sex composition of children is associated with the desire for an additional child. One aspect of this influence is the preference for sons. In India, relative to women with no daughters, women with no sons have significantly higher odds of progressing to the next birth (Rajan et al., 2018).

##### Mesosystems

The mesosystem comprises the reciprocal interactions that take place among the Microsystems within an individual's life. Although a couple is defined as a union system, the interaction between two partners is affected by other system interactions. This subsystem emphasizes the surrounding interpersonal relationships (Jones et al., 2011). The most frequent variables in a couple's mesosystem are marital status, parity, and partnership satisfaction. Gender role attitude (traditional/egalitarian) is another important component in this system. The mesosystem of childbearing intention also includes family and peers network which can influence couples' decisions.

#### 3.1.5 Marital status

Partnership stability has a clear significant role in the realization of childbearing intentions (Berninger et al., 2011; De Wachter & Neels, 2011; Goldscheider et al., 2013; Hanappi et al., 2017; Modena & Sabatini, 2012; Rosina & Testa, 2009; Spéder & Kapitány, 2009; Vignoli et al., 2013). Men prefer not to be alone and thus being a cohabitant or married increases their intentions to have a child. However, the partnership type (cohabitation or legal marriage) is especially important for women (Spéder & Kapitány, 2009). Legal married women are more likely to realize their childbearing intentions in a short time (De Wachter & Neels, 2011; Risse, 2010; Rosina & Testa, 2009; Spéder & Kapitány, 2009; Vignoli et al., 2013).

Single men and women have less tendency to have a child. The higher proportion of single non‐cohabitants men and women fail to realize their fertility intentions. (Timæus & Moultrie, 2020).

#### 3.1.6 Parity

Parity is an important criterion in childbearing decision‐making (Hanappi et al., 2017; Park et al., 2008). People with more children are more likely to abandon their intentions (De Wachter & Neels, 2011; Modena & Sabatini, 2012; Rijken & Liefbroer, 2009; Risse, 2010; Spéder & Kapitány, 2009).

#### 3.1.7 Partnership satisfaction

A satisfactory partnership should be desired with the intention to have a child. Positive partnership quality is associated with childbearing intention (Berninger et al., 2011; Rijken & Liefbroer, 2009). Women reporting intimate partner violence are less likely to desire more children (Kuhlmann et al., 2019).

#### 3.1.8 Gender role attitude

Gender‐role attitudes—including traditional and egalitarian attitudes (Kaufman, 2000; Miettinen et al., 2011; Vitali et al., 2009; Yoon, 2016), division of housework (Bernhardt et al., 2016; Fiori, 2011; Harknett et al., 2014; Mills et al., 2008; Rosina & Testa, 2009; Yoon, 2016; Yu & Kuo, 2017), childcare tasks (Fiori, 2011; Yoon, 2016) and perception of the division (De Wachter & Neels, 2011; Fiori et al., 2013) play major roles in childbearing decision‐making among couples. Women's satisfaction with sharing home chores increases the probability of their agreement to have a child (Dommermuth, et al., 2017; Goldscheider et al., 2013; Rosina & Testa, 2009).

Traditional men are more likely to have a child (Bernhardt et al., 2016; Kaufman, 2000). Egalitarian men seem to partner to a great extent than traditional men (Bernhardt et al., 2016). Egalitarian men are more likely to enter unions while traditional women are more likely to have a child. These findings showed that gender role attitudes have a different impact on fertility intentions among men and women (Kaufman, 2000).

#### 3.1.9 Family and peers network

Interaction within networks of family and friends is a major component in childbearing decision‐making (Kuhnt & Trappe, 2016). Informal family help can positively increase childbearing intention among women (Fiori, 2011; Fiori et al., 2013; Park et al., 2008; Schaffnit & Sear, 2017; Yu & Kuo, 2017).

### 3.2 Exosystems

The exosystem includes a situation or institution that affects an individual's daily settings but is not part of the individual's immediate environment (Rothbaum et al., 2002). The exosystem of the ECSM includes certain variables, such as job characteristics, urban residence and housing conditions.

#### 3.2.1 Job characteristic

The pattern of employment effect varies by gender and parity. Full‐time work is a key factor for childless men and women to become a parent shortly. For the second child, full‐time employment loses its positive influence on mothers (Neyer et al., 2013). Job insecurity could affect childbearing intention negatively. Job instability in low and medium‐educated people is less responsive compared with highly educated ones (Hanappi et al., 2017; Vignoli et al., 2020). Fiori et al., (2013) revealed that job insecurity is more associated with short‐term intentions, and the number of children intended by couples is less affected by it.

#### 3.2.2 Urban residence

Living in districts characterized by some forms of unease (high rate of school dropout, many minors followed by social services etc.) decreases childbearing intention (Meggiolaro, 2011; Riederer & Buber, 2019).

#### 3.2.3 Housing condition

Housing type is associated with childbearing intention. Homeowners and couples living in single‐family houses are significantly more likely to have their first child sooner (Kulu & Vikat, 2007; Park et al., 2008; Vignoli et al., 2013). Living in single‐family houses is associated with a higher intention to have a first child compared with living in apartments. (Kulu & Vikat, 2007; Park et al., 2008).

### 3.3 Macrosystem

The macrosystem of the ECSM comprises cultural and societal principles with broader influences on the couple's system.

#### 3.3.1 Family policies

A large body of fertility literature has concentrated on specific family policies and the degree of availability of childcare services (Fiori et al., 2013; Park & Cho, 2011; Wesolowski, 2015; Yu & Kuo, 2017). In developed societies, higher‐order births are likely to be more responsive to policy and environmental changes compared with the first births (Morgan & Taylor, 2006). Countries with family policies—as a part of their labour market policies, care policies and gender policies—seem to keep fertility above the lowest‐low levels (Wesolowski, 2015).

#### 3.3.2 Religiosity

Religiosity also plays an important role in childbearing decision‐making. Religious couples are more likely to become parents (Kuhnt & Trappe, 2016; Rosina & Testa, 2009). But, the religiousness of only one of the two partners will cause conflict between couples. The results showed that when a woman wants a child but, the man does not, male religiosity increases the opposition. In contrast, when the man wants a child but the woman does not, women's religiosity decreases opposition between the couple (Rosina & Testa, 2009). Belief in God is independently associated with fertility desires. At least some of the connections between religiosity and fertility is attributed to metaphysical beliefs—and not just traditional and institutional religiosity (Cranney, 2015).

#### 3.3.3 Child value

The value placed on having children can motivate fertility intentions. Psychological benefits (e.g. comfort and paternal and maternal feelings) of the child show a greater preference for a second child's intention. Instrumental values (e.g. current economic support and in the elderly years, a continuation of the family line and social duty) of children are not significantly associated with the intentions to have a child (Miettinen et al., 2011; Park & Cho, 2011).

#### 3.3.4 Individualism attitudes

Self‐realization is a predictor that affects childbearing intentions negatively. Nowadays, people believe that more children cannot increase their social esteem enough to take charge of raising a child (Wesolowski, 2015).

### 3.4 Chronosystem

The chronosystem of the ECSM consists of changes relating to time or throughout the lifecycle that influence individuals and their environment (Bronfenbrenner, 1986). For example, when a couple's lives are shared and merged, their chronosystems are also shared and merged. The chronosystem continuously changes and evolves. Couples prefer to postpone their childbearing intentions in the early years of the marriage. In general, pregnancy desire increased over time as a relationship endured and became more serious (Barbe et al., 2019).

Increasing female labour force participation was initially related to the negative effect on fertility rate in developed countries. The expansion of modernity and men's participation in family chores turned it positive since the 1990s.

## 4 DISCUSSION

Childbearing intentions are not always voluntary and are often influenced by actual and perceived circumstances (Holton et al., 2011).

Childbirth in the early years of the marriage sounds like a threat for couples to spend their time together. Losing their freedom, leisure time and traveling opportunities would lead them to delay childbearing. Over time in some couples, this attitude may change and the need for children is created (Qu & Weston, 2001). As one grows older, his/her time left for childbearing decreases. However, the biological limits are known for women, but social constraints apply to both men and women in childbearing decision‐making (Spéder & Kapitány, 2009).

First‐birth intentions are closely related to the wish of establishing a family and more influenced by normative pressure than the economic situation. Second‐birth intentions are also governed by age but related to educational level, availability and cost of childcare and labour market situation (Wesolowski, 2015).

Education can simultaneously indicate the economic and cultural effect mechanisms. A variety of lifestyles and cultural resources are tied up with education. (Fiori et al., 2013; Spéder & Kapitány, 2009) Therefore, the interaction will be created not only within the couple but also between the couple and other systems.

Higher education level is positively associated with higher fertility intention. It could be due to a lack of resources among those with lower education (Brauner‐Otto & Geist, 2018; Mills et al., 2008), as well as higher advantageous positions among high‐level educated ones (Dommermuth, et al., 2017; Wesolowski, 2015). In countries where there are more opportunities for women to reach high levels of education, other structural circumstances affecting fertility are also available, for example life satisfaction, sense of well‐being and levels of trust. Besides, policies can successfully combine work and family life for highly educated women in these countries. Undoubtedly, the marriage market has also an important role in this regard. As highly educated women have more opportunities to marry and have a better‐educated partner, so they can plan to have larger families (Testa et al., 2011).

But in some societies, educated women increasingly face a deficit of educated men with whom to pursue childbearing. This leads them to resort to elective egg freezing (EEF). Women may be resorting to EEF to pursue careers and achieve reproductive autonomy (Inhorn et al., 2018).

Although non‐working women have enough time to raise children, lack of personal investments in non‐working women may let them enjoy less fortunate conditions and also receive less support from their partner and family, and institutions. As a result, it may limit their intentions to have a child (Wesolowski, 2015). The psychological value of children, such as providing comfort during old age, is associated with higher intentions to have a second child among non‐working mothers. Non‐working mothers may be more dependent on their children for emotional support during their elderly years compared with employed women and even fathers. The labour force participation of women may provide an alternative source of satisfaction, which can decrease the psychological advantages of children (Park & Cho, 2011). There is an indirect effect of satisfaction with job security on childbearing intention. More satisfaction with job security may lead to fewer conflicts in the couple relationship. Hence, it may lead to satisfaction within a partnership and a higher intention to have a first child (Dommermuth et al., 2017).

The results showed that happier men and women prefer to become parents sooner. When both the woman and the man report a particularly high level of happiness, the probability of becoming parents for the first time increases more than when only one of the two partners is happier than usual. (Timæus & Moultrie, 2020). Women with a low level of happiness might not have a positive experience with the first child while women with a high level of happiness may not want to change the positive status they live; So both prefer to limit their childbearing intentions (Aassve et al., 2016). Health concerns can seriously affect second child intentions among couples. Poor health threatens childbirth and may compromise the health of both mother and child (Wesolowski, 2015).

Partnership stability is an important factor in the transition to parenthood for both men and women. So, single men and women have less tendency to have a child (De Wachter & Neels, 2011; Modena & Sabatini, 2012; Risse, 2010; Rosina & Testa, 2009; Spéder & Kapitány, 2009; Vignoli et al., 2013); however, differences between legally married couples and cohabitant couples depend on the norms of each society (Dommermuth, et al., 2017; Kuhnt & Trappe, 2016; Modena & Sabatini, 2012).

In zero parity, a couple agreement or disagreement has the same outcome. As childlessness is not a norm in most countries, under the pressure of society, they will experience a birth almost as often as the same (Testa et al., 2011). The possibility for an additional child is associated with couples’ desires. As parenthood is more related to women's lives, it seems that the female partner's opinion plays an essential role in childbirth decision‐making (Bernhardt et al., 2016; Fan & Maitra, 2010). When a man wants to have a child but, his wife does not, he prefers to express negative or uncertain childbearing intention. It indicates that women have a stronger influence on short‐term childbearing intentions (Bernhardt et al., 2016). It is undeniable that the man's active involvement in childcare duties turns disagreement more towards childbearing in higher parities (Testa et al., 2011).

Although a state of satisfactory partnerships is required to have a child (Fiori et al., 2013), both highly positive and highly negative interactions between couples have a significant negative influence on the rates of the first and subsequent births. A great deal of satisfaction means partners are happy with their current family situation so having another child may threaten their satisfactory condition. Unsatisfactory relationships between couples disturb a suitable pre‐condition for child‐rearing. Thus, an additional child may face their life with a new challenge. Nevertheless, effective aspects of relationship quality and their mechanisms are still unknown (Park et al., 2008).

Gender role attitudes have an important effect on how men and women view parenthood (Yoon, 2016; Yu & Kuo, 2017). Traditional men are more likely to partner with traditional women. In comparison, egalitarian men might prefer to partner with egalitarian women who are considerably less fascinated by the benefits of motherhood. Holding a traditional gender role attitude is positively related to childbearing (Bernhardt et al., 2016; Dommermuth, et al., 2017). In comparison, egalitarian men are more likely to partner and remain partnered (Bernhardt et al., 2016). It seems that they are more attractive to their partners as they share more equally with them. Having less conflict with their wives leads to a happier marital relationship and may encourage them to plan to have a child. That is why most women prefer men who share in the responsibilities of the household (Kaufman, 2000).

Childless couples are influenced by network partners and their family size. Unlike one‐child peers, peers with two or more children have a negative influence on the intentions of the childless couples. Women are more likely to have a/another child while they live with or near parents (in‐law). Also, this behaviour is established among those who are not working or whose husbands work relatively long hours or have relatively low educational attainment (Raymo et al., 2010). However, frequent intergeneration exchange and co‐residence decrease intentions to have an extra child (Harknett et al., 2014).

Although strong extended family ties are expected to encourage higher levels of fertility, the generation of middle‐aged adults (so‐called, “sandwich generation”) may face concurrent commitment to support elderly parents and dependent children (Grundy & Henretta, 2006; Testa et al., 2011). Therefore, they may limit their family size to limit their support obligations (Harknett et al., 2014). In another way, couples from large families have experiences of having probable problems related to a large number of siblings. Their parents may evaluate their own high fertility experiences negatively and lead them to limit their fertility intentions (Bühler & Fratczak, 2007).

Living in single‐family houses is associated with a higher intention to have a first child compared with living in apartments. This result may be due to the impossibility of expanding an apartment space or living in crowded apartments. However, the exact reasons are unclear (Kulu & Vikat, 2007; Park et al., 2008). In countries with no government regulation in the house renting market, couples face a difficult housing regime (Kulu & Vikat, 2007; Vignoli et al., 2013).

Religion and family make up a large proportion of shaping personal identity. Higher fertility intention among religious people is related to family beliefs and values, including schemas about the importance of marriage and parenthood, and gender roles in families. Fertility differentials are a part of a widespread association between religiosity and family behaviour (Hayford & Morgan, 2008).

Social pressure has more influence on personal goals formation (Kuhnt & Trappe, 2016; Meggiolaro, 2011). Parents are the main part of the communication network (Bühler & Fratczak, 2007; Kuhnt & Trappe, 2016). Moreover, the pressure exerted from parents is more effective than friends (Kuhnt & Trappe, 2016).

According to ECSM, fertility behaviour largely depends on the characteristics and ability of individuals to communicate experiences and information (Bühler & Fratczak, 2007). Influential communication networks show reproductive planning and decision‐making (Fan & Maitra, 2010; Kuhnt & Trappe, 2016; Testa, 2012; Vignoli et al., 2013). Kuhnt and Trappe, (2016) mentions that those perceived social pressure to have a child are more likely to have positive fertility intentions and vice versa. This may reflect the willingness of individuals to comply with norms.

Increasing female labour force participation in developed countries around the world was initially associated with a decline in the fertility rate. Gender inequality and the difficulty of combining work and family are possible explanations for this. However, the negative relationship between women's labour force participation and childbearing has turned positive since 1990 (Averett & Whittington, 2001; Bernhardt et al., 2016; Mills et al., 2008).

In developed societies, higher‐order births are likely to be more responsive to policy and environmental changes compared with the first births (Morgan & Taylor, 2006). Labour market policies are expected to change the labour market so that couples can maintain their employment and income, even with young children (Neyer et al., 2013; Wesolowski, 2015). Countries with high national‐level gender equality also need household‐level equity to see an increase in fertility (Bernhardt et al., 2016; Dommermuth, et al., 2017; Mynarska et al., 2015).

## 5 CONCLUSION

Our analysis demonstrates that childbearing intention is affected by complex reciprocal interactions of multilevel ecological factors that can help policymakers expand their awareness of factors that affect fertility. Hence, it is not possible to separate the influence of one domain from another. To promote childbirth, it is essential to consider a multidimensional programme according to the features of each regional and geographic area.

## 6 STUDY STRENGTHS AND LIMITATIONS

53 studies met our inclusion criteria. 24 (45%) of studies were conducted on both men and women, 27 (51%) on women and just 2 (4%) on men. Most of the studies deal exclusively with women, and the theorizing regarding what leads to childbearing decision‐making, particularly among men, is almost underdeveloped.

Fertility choices have been studied mostly from an individual view. The limited research and data existing on couples restrict our information about fertility intentions.

There are limited instances of interdisciplinary studies, studying fertility would highly benefit by crossing disciplinary and geographic boundaries.

Developing comparable data collection in many countries will improve fertility research. The lack of actual instruments such as the collection of quantitative data for the network‐based approach is another key factor in infertility studies.

## CONFLICT OF INTERESTS

Not applicable.

## AUTHORS’ CONTRIBUTIONS

M. H, M Sh, A N and AK: Conception and Design. MH and MSH: Acquisition of Data. MH, MSH, AN and AK: Analysis and Interpretation of Data. MH and MSH: Drafting the Article. MH, MSH, AN and AK: Revising It for Intellectual Content.

## Supporting information

Supplementary Material

## Data Availability

The data that support the findings of this study are available from the corresponding author, upon reasonable request.
