# Supplementary material for: Childbearing intention and its associated factors: A systematic review
Source: Nurs Open. 2021 Mar 11;8(5):2354–68. doi: 10.1002/nop2.849 (PMC8363403; doi:10.1002/nop2.849)
Supplement: Supplementary file 1 — Supplementary Material [file NOP2-8-2354-s001.docx]

**Appendix1.search strategy**

| 1 | Reproductive decision making. mp. | **Ovid MEDLINE** |
| --- | --- | --- |
| 2 | ((pregnan$ or fertili$ or reproductiv$ or childbearing) adj (intent$ or intend$ or want$ or desire$)).mp. |  |
| 3 | 1 or 2 |  |
| 4 | HIV.ti. |  |
| 5 | Cancer$.ti. |  |
| 6 | "Human immunodeficiency virus".ti. |  |
| 7 | 4 or 5 or 6 |  |
| 8 | 3 not 7 |  |
| 9 | limit 8 to English |  |
| 1 | Reproductive decision making. mp. | **EMBASE** |
| 2 | ((pregnan$ or fertili$ or reproductiv$ or childbearing) adj (intent$ or intend$ or want$ or desire$)).mp. |  |
| 3 | 1 or 2 |  |
| 4 | HIV.ti. |  |
| 5 | Cancer$.ti. |  |
| 6 | "Human immunodeficiency virus".ti. |  |
| 7 | 4 or 5 or 6 |  |
| 8 | 3 not 7 |  |
| 9 | limit 8 to exclude MEDLINE journals |  |
| 10 | limit 9 to the English language |  |
| 1 | Reproductive decision making. mp. | **PsycINFO** |
| 2 | ((pregnan$ or fertili$ or reproductiv$ or childbearing) adj (intent$ or intend$ or want$ or desire$)).mp. |  |
| 3 | 1 or 2 |  |
| 4 | HIV.ti. |  |
| 5 | Cancer$.ti. |  |
| 6 | "Human immunodeficiency virus".ti. |  |
| 7 | 4 or 5 or 6 |  |
| 8 | 3 not 7 |  |
| 9 | limit 8 to English language |  |
| 1 | TX "reproductive decision making" | **CINAHL** |
| 2 | TX ((pregnan* or fertili* or reproductiv* or childbearing) W1 (intent* or intend* or want* or desire*)) |  |
| 3 | 1 or 2 |  |
| 4 | TI HIV |  |
| 5 | TI cancer* |  |
| 6 | TI "Human immunodeficiency virus" |  |
| 7 | 4 or 5 or 6 |  |
| 8 | 3 not 7 |  |
| 9 | limit 8 to English language |  |
| 1 | TS=("reproductive decision making") | **Web of Science** |
| 2 | TS=((pregnan* OR fertili* OR reproductiv* OR childbearing) NEAR/1 (intent* OR intend* OR want* OR desire*)) |  |
| 3 | 1 or 2 |  |
| 4 | TI=HIV |  |
| 5 | TI=cancer* |  |
| 6 | TI="Human immunodeficiency virus" |  |
| 7 | 4 or 5 or 6 |  |
| 8 | 3 not 7 |  |
| 9 | limit 8 to English language |  |
|  | ( ( TITLE-ABS-KEY ( "reproductive decision making" ) ) OR ( TITLE-ABS-KEY ( ( pregnan* OR fertili* OR reproductiv* OR childbearing ) PRE/2 ( intent* OR intend* OR want* OR desire* ) ) ) ) AND NOT ( ( TITLE ( hiv ) ) OR ( TITLE ( cancer* ) ) OR ( TITLE ( "Human immunodeficiency virus" ) ) ) AND NOT INDEX ( medline ) AND ( LIMIT-TO ( SRCTYPE , "j " ) OR LIMIT-TO ( SRCTYPE , " p " ) ) AND ( LIMIT-TO ( LANGUAGE , "English " ) ) | **Scopus** |

**Appendix 2.Quality Assessment of Included Studies**

| Author | Year | Title and Abstract | Introduction/2 | | Methods/12 | | | | | | | | | Results/9 | | | | | Discussion/5 | | | |  |  | | Quality rating |
| --- | --- | --- | --- | --- | --- | --- | --- | --- | --- | --- | --- | --- | --- | --- | --- | --- | --- | --- | --- | --- | --- | --- | --- | --- | --- | --- |
|  |  |  | Background | Objectives | Study Design | Setting | Participants | Variables | Data sources/measurement | Bias | Study size | Quantitative variables | Statistical methods | Participants | Descriptive data | Outcome data | Main results | Other analyses | Key results | Limitations | Interpretation | Generalizability | Funding/1 | | Total score |  |
| Aassve A | 2016 | 2 | 1 | 1 | 1 | 1 | 1 | 1 | 1 | 0 | 1 | 1 | 2 | 1 | 1 | 1 | 3 | 1 | 1 | 1 | 1 | 1 | 1 | | 25 | High |
| Averett, S. L | 2001, | 2 | 1 | 1 | 1 | 1 | 2 | 1 | 1 | 0 | 1 | 1 | 2 | 1 | 1 | 1 | 1 | 1 | 1 | 0 | 1 | 1 | 1 | | 23 | Moderate |
| Barber, J. | 2019 | 2 | 1 | 1 | 1 | 1 | 2 | 1 | 1 | 0 | 1 | 1 | 2 | 1 | 1 | 1 | 2 | 1 | 1 | 1 | 1 | 1 | 1 | | 25 | High |
| Bernhardt E | 2016 | 2 | 1 | 1 | 1 | 1 | 2 | 1 | 1 | 0 | 1 | 1 | 3 | 1 | 1 | 1 | 4 | 1 | 1 | 1 | 1 | 1 | 1 | | 28 | High |
| Berninger, I. | 2011 | 1 | 1 | 1 | 1 | 1 | 2 | 1 | 1 | 0 | 1 | 1 | 3 | 1 | 1 | 1 | 2 | 1 | 1 | 0 | 1 | 1 | 1 | | 24 | High |
| Boivin, J | 2017 | 2 | 1 | 1 | 1 | 1 | 2 | 1 | 1 | 1 | 1 | 1 | 2 | 1 | 1 | 1 | 2 | 1 | 1 | 1 | 1 | 1 | 1 | | 26 | High |
| Bühler, C. | 2007 | 2 | 1 | 1 | 1 | 1 | 2 | 1 | 1 | 0 | 1 | 1 | 2 | 1 | 1 | 1 | 2 | 1 | 1 | 0 | 1 | 1 | 1 | | 24 | High |
| Brauner S R | 2017 | 2 | 1 | 1 | 1 | 1 | 2 | 1 | 1 | 0 | 1 | 1 | 3 | 1 | 1 | 1 | 3 | 1 | 1 | 0 | 1 | 1 | 1 | | 27 | High |
| Cranney, S. | 2015 | 2 | 1 | 1 | 1 | 1 | 2 | 1 | 1 | 0 | 1 | 1 | 2 | 1 | 1 | 1 | 2 | 1 | 1 | 0 | 1 | 1 | 1 | | 23 | Moderate |
| De Wachter, | 2011 | 2 | 1 | 1 | 1 | 1 | 2 | 1 | 1 | 0 | 1 | 1 | 2 | 1 | 1 | 1 | 3 | 1 | 1 | 0 | 1 | 1 | 1 | | 25 | High |
| Dommermut | 2015 | 2 | 1 | 1 | 1 | 1 | 2 | 1 | 1 | 0 | 1 | 1 | 3 | 1 | 1 | 1 | 2 | 1 | 1 | 0 | 1 | 1 | 1 | | 25 | High |
| Fan E | 2012 | 1 | 1 | 1 | 1 | 1 | 2 | 1 | 1 | 0 | 1 | 1 | 3 | 1 | 1 | 1 | 2 | 1 | 1 | 0 | 1 | 1 | 1 | | 24 | High |
| Fahlén S | 2013 | 2 | 1 | 1 | 1 | 1 | 2 | 1 | 1 | 0 | 1 | 1 | 3 | 1 | 1 | 1 | 2 | 1 | 1 | 0 | 1 | 1 | 1 | | 25 | High |
| Fiori F | 2011 | 2 | 1 | 1 | 1 | 1 | 2 | 1 | 1 | 0 | 1 | 1 | 3 | 1 | 1 | 1 | 2 | 1 | 1 | 0 | 1 | 1 | 1 | | 25 | High |
| Fiori F | 2013 | 2 | 1 | 1 | 1 | 1 | 2 | 1 | 1 | 0 | 1 | 1 | 3 | 1 | 1 | 1 | 3 | 1 | 1 | 0 | 1 | 1 | 1 | | 26 | High |
| Goldscheider | 2013 | 2 | 1 | 1 | 1 | 1 | 2 | 1 | 1 | 0 | 1 | 1 | 2 | 1 | 1 | 1 | 3 | 1 | 1 | 0 | 1 | 1 | 1 | | 25 | High |
| Hanappi, D. | 2017 | 2 | 1 | 1 | 1 | 1 | 2 | 1 | 1 | 0 | 1 | 1 | 3 | 1 | 1 | 1 | 3 | 1 | 1 | 1 | 1 | 1 | 1 | | 27 | High |
| Harknett, K | 2014 | 2 | 1 | 1 | 1 | 1 | 2 | 1 | 1 | 0 | 1 | 1 | 2 | 1 | 1 | 1 | 2 | 1 | 1 | 0 | 1 | 1 | 1 | | 24 | High |
| Kaufman, G. | 2000 | 1 | 1 | 1 | 1 | 1 | 2 | 1 | 1 | 0 | 1 | 1 | 2 | 1 | 1 | 1 | 2 | 1 | 1 | 0 | 1 | 1 | 1 | | 23 | Moderate |
| Hayford,S R | 2008 | 1 | 1 | 1 | 1 | 1 | 2 | 1 | 1 | 0 | 1 | 1 | 2 | 1 | 1 | 1 | 3 | 1 | 1 | 0 | 1 | 1 | 1 | | 24 | High |
| Kjerulff K | 2013 | 2 | 1 | 1 | 1 | 1 | 2 | 1 | 1 | 0 | 1 | 1 | 3 | 1 | 1 | 1 | 4 | 1 | 1 | 1 | 1 | 1 | 1 | | 29 | High |
| Kim, E. H. W. | 2017 | 2 | 1 | 1 | 1 | 1 | 2 | 1 | 1 | 0 | 1 | 1 | 3 | 1 | 1 | 1 | 3 | 1 | 1 | 0 | 1 | 1 | 1 | | 26 | High |
| Kuhlmann, | 2019 | 2 | 1 | 1 | 1 | 1 | 2 | 1 | 1 | 0 | 1 | 1 | 2 | 1 | 1 | 1 | 2 | 1 | 1 | 1 | 1 | 1 | 1 | | 25 | High |
| Kuhnt, A. K. | 2016 | 2 | 1 | 1 | 1 | 1 | 2 | 1 | 1 | 0 | 1 | 1 | 3 | 1 | 1 | 1 | 3 | 1 | 1 | 0 | 1 | 1 | 1 | | 26 | High |
| Kulu Hill. | 2007 | 2 | 1 | 1 | 1 | 1 | 2 | 1 | 1 | 0 | 1 | 1 | 3 | 1 | 1 | 1 | 2 | 1 | 1 | 0 | 1 | 1 | 1 | | 25 | High |
| Meggiolaro S | 2011 | 1 | 1 | 1 | 1 | 1 | 2 | 1 | 1 | 0 | 1 | 1 | 2 | 1 | 1 | 1 | 2 | 1 | 1 | 0 | 1 | 1 | 1 | | 23 | Moderate |
| Miettinen, A. | 2011 | 2 | 1 | 1 | 1 | 1 | 2 | 1 | 1 | 0 | 1 | 1 | 3 | 1 | 1 | 1 | 2 | 1 | 1 | 1 | 1 | 1 | 1 | | 26 | High |
| Metcalfe, A. | 2014 | 2 | 1 | 1 | 1 | 1 | 2 | 1 | 1 | 0 | 1 | 1 | 3 | 1 | 1 | 1 | 3 | 1 | 1 | 1 | 1 | 1 | 1 | | 27 | High |
| Mills M, | 2008 | 2 | 1 | 1 | 1 | 1 | 2 | 1 | 1 | 0 | 1 | 1 | 3 | 1 | 1 | 1 | 3 | 1 | 1 | 0 | 1 | 1 | 1 | | 27 | High |
| Modena,F. | 2012 | 2 | 1 | 1 | 1 | 1 | 2 | 1 | 1 | 0 | 1 | 1 | 3 | 1 | 1 | 1 | 2 | 1 | 1 | 0 | 1 | 1 | 1 | | 25 | High |
| Mynarska, M | 2020 | 2 | 1 | 1 | 1 | 1 | 2 | 1 | 1 | 0 | 1 | 1 | 2 | 1 | 1 | 1 | 2 | 1 | 1 | 0 | 1 | 1 | 0 | | 23 | Moderate |
| Neyer, et al. | 2013 | 2 | 1 | 1 | 1 | 1 | 2 | 1 | 1 | 0 | 1 | 1 | 3 | 1 | 1 | 1 | 2 | 1 | 1 | 0 | 1 | 1 | 1 | | 25 | High |
| Park, S. M. | 2008 | 2 | 1 | 1 | 1 | 1 | 3 | 1 | 1 | 0 | 1 | 1 | 2 | 1 | 1 | 1 | 4 | 1 | 1 | 0 | 1 | 1 | 1 | | 27 | High |
| Park, S. M | 2011 | 2 | 1 | 1 | 1 | 1 | 2 | 1 | 1 | 0 | 1 | 1 | 3 | 1 | 1 | 1 | 4 | 1 | 1 | 0 | 1 | 1 | 1 | | 28 | High |
| Preis, H | 2020 | 2 | 1 | 1 | 1 | 1 | 2 | 1 | 1 | 0 | 1 | 1 | 2 | 1 | 1 | 1 | 3 | 1 | 1 | 1 | 1 | 1 | 0 | | 25 | High |
| Rajan, S | 2018 | 2 | 1 | 1 | 1 | 1 | 2 | 1 | 1 | 1 | 1 | 1 | 2 | 1 | 1 | 1 | 2 | 1 | 1 | 0 | 1 | 1 | 1 | | 25 | High |
| Raymo, J. M. | 2010 | 2 | 1 | 1 | 1 | 1 | 2 | 1 | 1 | 0 | 1 | 1 | 3 | 1 | 1 | 1 | 2 | 1 | 1 | 1 | 1 | 1 | 1 | | 26 | High |
| Riederer, B | 2019 | 1 | 1 | 1 | 1 | 1 | 2 | 1 | 1 | 0 | 1 | 1 | 2 | 1 | 1 | 1 | 3 | 1 | 1 | 0 | 1 | 1 | 1 | | 24 | High |
| Rijken Æ | 2009 | 2 | 1 | 1 | 1 | 1 | 1 | 1 | 1 | 0 | 1 | 1 | 2 | 1 | 1 | 1 | 2 | 1 | 1 | 0 | 1 | 1 | 1 | | 22 | Moderate |
| Risse, L | 2010 | 2 | 1 | 1 | 1 | 1 | 2 | 1 | 1 | 1 | 1 | 1 | 2 | 1 | 1 | 1 | 3 | 1 | 1 | 0 | 1 | 1 | 1 | | 27 | High |
| Rosina, A. | 2009 | 2 | 1 | 1 | 1 | 1 | 1 | 1 | 1 | 0 | 1 | 1 | 2 | 1 | 1 | 1 | 3 | 1 | 1 | 1 | 1 | 1 | 1 | | 25 | High |
| Schaffnit, S. | 2017 | 2 | 1 | 1 | 1 | 1 | 2 | 1 | 1 | 0 | 1 | 1 | 3 | 1 | 1 | 1 | 2 | 1 | 1 | 0 | 1 | 1 | 1 | | 25 | High |
| Spéder, Z. | 2009 | 2 | 1 | 1 | 1 | 1 | 2 | 1 | 1 | 0 | 1 | 1 | 2 | 1 | 1 | 1 | 2 | 1 | 1 | 0 | 1 | 1 | 1 | | 24 | High |
| Sinyavskaya | 2013 | 1 | 1 | 1 | 1 | 1 | 2 | 1 | 1 | 0 | 1 | 1 | 3 | 1 | 1 | 1 | 3 | 1 | 1 | 0 | 1 | 1 | 1 | | 24 | High |
| Testa, M. R., | *2011* | 2 | 1 | 1 | 1 | 1 | 2 | 1 | 1 | 0 | 1 | 1 | 3 | 1 | 1 | 1 | 3 | 1 | 1 | 0 | 1 | 1 | 1 | | 27 | High |
| Testa, M. R., | 2012 | 2 | 1 | 1 | 1 | 1 | 2 | 1 | 1 | 0 | 1 | 1 | 3 | 1 | 1 | 1 | 2 | 1 | 1 | 0 | 1 | 1 | 1 | | 25 | High |
| Testa, M. R | 2014 | 2 | 1 | 1 | 1 | 1 | 2 | 1 | 1 | 0 | 1 | 1 | 2 | 1 | 1 | 1 | 2 | 1 | 1 | 0 | 1 | 1 | 1 | | 24 | High |
| Vignoli, D | 2020 | 1 | 1 | 1 | 1 | 1 | 2 | 1 | 1 | 0 | 1 | 1 | 3 | 1 | 1 | 1 | 3 | 1 | 1 | 0 | 1 | 1 | 1 | | 25 | High |
| Vignoli, D. | 2012 | 2 | 1 | 1 | 1 | 1 | 2 | 1 | 1 | 0 | 1 | 1 | 2 | 1 | 1 | 1 | 2 | 1 | 1 | 1 | 1 | 1 | 1 | | 25 | High |
| Vitali, A. | 2009 | 1 | 1 | 1 | 1 | 1 | 1 | 1 | 1 | 0 | 1 | 1 | 1 | 1 | 1 | 1 | 1 | 1 | 1 | 0 | 1 | 1 | 1 | | 20 | Moderate |
| Wesolowski, K | 2015 | 1 | 1 | 1 | 1 | 1 | 2 | 1 | 1 | 1 | 1 | 1 | 3 | 1 | 1 | 1 | 3 | 1 | 1 | 0 | 1 | 1 | 1 | | 25 | High |
| Yoon, S. Y | 2016 | 2 | 1 | 1 | 1 | 1 | 2 | 1 | 1 | 0 | 1 | 1 | 3 | 1 | 1 | 1 | 3 | 1 | 1 | 1 | 1 | 1 | 1 | | 28 | High |
| Yu, W. H. | 2017 | 2 | 1 | 1 | 1 | 1 | 2 | 1 | 1 | 0 | 1 | 1 | 3 | 1 | 1 | 1 | 2 | 1 | 1 | 0 | 1 | 1 | 1 | | 25 | High |
